# Non-Target Site Mechanisms of Fungicide Resistance in Crop Pathogens: A Review

**DOI:** 10.3390/microorganisms9030502

**Published:** 2021-02-27

**Authors:** Mengjun Hu, Shuning Chen

**Affiliations:** 1 Department of Plant Science and Landscape Architecture, University of Maryland, College Park, MD 20742, USA; 2 Institute of Plant Protection, Chinese Academy of Agricultural Sciences, Beijing 100193, China

**Keywords:** fungicide resistance, molecular basis, non-target site, plant pathogens

## Abstract

The rapid emergence of resistance in plant pathogens to the limited number of chemical classes of fungicides challenges sustainability and profitability of crop production worldwide. Understanding mechanisms underlying fungicide resistance facilitates monitoring of resistant populations at large-scale, and can guide and accelerate the development of novel fungicides. A majority of modern fungicides act to disrupt a biochemical function via binding a specific target protein in the pathway. While target-site based mechanisms such as alternation and overexpression of target genes have been commonly found to confer resistance across many fungal species, it is not uncommon to encounter resistant phenotypes without altered or overexpressed target sites. However, such non-target site mechanisms are relatively understudied, due in part to the complexity of the fungal genome network. This type of resistance can oftentimes be transient and noninheritable, further hindering research efforts. In this review, we focused on crop pathogens and summarized reported mechanisms of resistance that are otherwise related to target-sites, including increased activity of efflux pumps, metabolic circumvention, detoxification, standing genetic variations, regulation of stress response pathways, and single nucleotide polymorphisms (SNPs) or mutations. In addition, novel mechanisms of drug resistance recently characterized in human pathogens are reviewed in the context of nontarget-directed resistance.

## 1. Introduction 

Fungicide use is a major component in the integrated disease management for crop production, especially in open fields. The estimated amount of fungicides and bactericides used worldwide increased from 393 to 530 million kilograms during 1990 to 2018 (Food and Agriculture Organization of the United Nations; http://www.fao.org/). Despite the availability of a wide variety of fungicides, control of major diseases heavily relies on only a few chemical classes due to efficacy or residue concerns [1,2], leading to a high pressure of resistance selection. Fungal pathogens hitchhiking on goods or latently infected in plants can easily be distributed between regions and countries, further challenging the resistance management. The rate of emergence of resistant fungal populations has been continuously rising globally since the 1980s, posing a serious threat to farming sustainability and profitability [3]. One way to cope with fungicide resistance is through fundamental understanding of its mechanisms, which can facilitate resistance monitoring and aid in the development of novel fungicides.

Numerous studies have been conducted in the past to characterize modes of action of fungicides and corresponding resistance mechanisms. Fungicides, in particular those with single sites of action, function to block specific enzymes in critical biological pathways. Thus, the change either in the structure or the expression level of the enzyme can largely affect fungicide efficacy. These target site-based resistance mechanisms have been found in many fungi with resistance to various fungicides belonging to different chemical classes [4]. While the structural change of target sites is typically due to mutations in residues involved in the binding site, overexpression of the target sites oftentimes results from the rearrangement or mutations in its promoter region [4,5,6]. In either case, the resistance is considered to be linked to a single genetic locus and heritable, and is named qualitative resistance. The level of resistance is largely determined by specific mutations or the expression level of a given target site [5,7]. In contrast, resistance can also be attributed to other factors or mutations in multiple loci, termed quantitative resistance [8]. In addition, other terms such as innate or natural resistance and epigenetic resistance are used to describe certain fungal species that are insensitive to a given fungicide and transient resistance mediated by gene regulation, respectively [9,10]. However, some of these alternative mechanisms of resistance have not been well studied, although field-resistant isolates that lacked mutations or had similar expression levels of target sites compared to sensitive isolates have been reported across pathosystems [9,11,12,13,14]. 

The recent advancements in genome sequencing, bioinformatics, and gene manipulation have greatly improved our capability to address limiting factors in understanding resistant genotypes beyond target sites. The purpose of this review was to summarize the known mechanisms that are not linked to target sites, in the hope of inspiring more efforts into the exploration of unknown molecular bases of fungicide resistance. Specifically, alternative mechanisms involving drug efflux transporters, standing genetic variations, metabolic breakdown and circumvention, SNPs, RNAi-based epimutation, as well as mutator genotypes were discussed. 

## 2. Resistance Mediated by Drug Efflux Transporters

Many studies have shown associations between enhanced activity of efflux transporters and emergence of resistance in a wide range of fungal pathogens [15,16,17,18], indicating efflux transporters may have a common and critical role in fungicide sensitivity. In addition, simultaneous resistance to multiple chemical classes of fungicides was found to be attributable to overexpression of efflux pumps in some important fungal pathogens. In this section, the general role of drug efflux transporters and the key members that have been linked to fungicide resistance were reviewed. 

### 2.1. Drug Efflux Transporter in Fungi

In all living organisms, drug efflux transporters are integral membrane-bound proteins that transport a wide range of substrates, such as protein macromolecules, ions, or small molecules across a biological membrane [19]. Thus, they can mediate the efflux of a variety of toxic substrates from the cell, preventing toxin accumulation. In fungi, toxins can be self-produced or come from the environment. Synthetic fungicides are typically regarded as toxins to fungal populations, of which the efficacy can therefore be affected by the activity of drug efflux transporter. Two major groups of drug transporters have been characterized in fungi, including ATP-binding cassette (ABC) transporters and major facilitator superfamily (MFS) transporters. 

**Figure 1:**
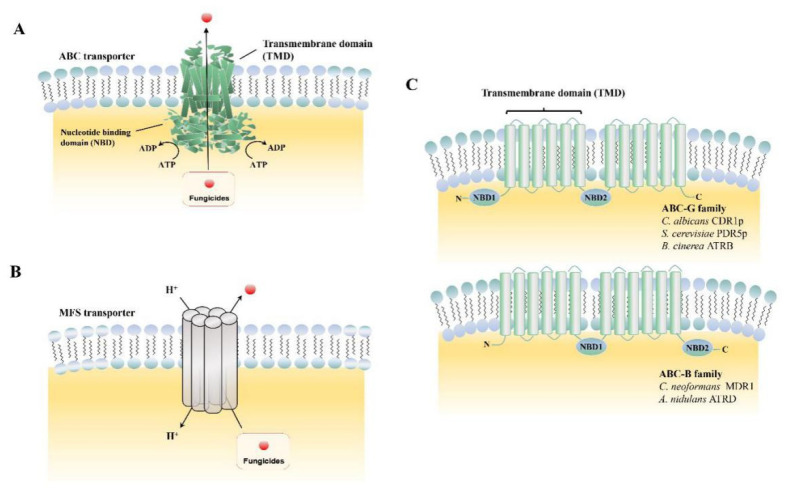
Biological structures of ATP-binding cassette (ABC) and major facilitator superfamily (MFS) transporters. (**A**) Domain arrangements of ABC transporters in the plane of the membrane; (**B**) working model of MFS transporter in the plane of the membrane; (**C**) schematic of “normal” arrangement of transmembrane domain (TMD), nucleotide-binding domain (NBD): Example of the ABC-G and ABC-B families.

The ABC superfamily is the dominant drug transporter, consisting of a transmembrane domain (TMDs) and a structurally conserved nucleotide-binding domain (NBDs) (Figure 1A,C). One important property of ABC transporters is their low substrate specificity, which allows them to transport a variety of structurally different compounds [20]. Based on the “architectures” of the protein, the ABC transporters were grouped into seven sub families (A–G). Unlike ABC transporters that export toxic compounds by tight coupling of ATP cleavage, the MFS transporters transfer compounds by the proton-motive force across the fungal plasma membrane (Figure 1B). In total, the MFS transporters contain 74 families, with each usually responsible for a specific substrate type [21]. Table 1 lists the ABC and MFS transporters currently identified in phytopathogenic fungi. The known functions of MFS transporters include drug efflux systems, transfer endogenously produced toxins, metabolites of the Krebs cycle, organophosphate/phosphate exchangers, and bacterial aromatic permeases [19].

### 2.2. ABC/MFS Genes in Plant Pathogenic Fungi

Overall, ascomycetes, zygomycetes, and basidiomycetes tend to have a significantly smaller number of ABC superfamily proteins and MFS transporters, compared to oomycetes and ancient Chytridiomycota [52]. In ascomycetes, approximately 40 to 50 ABC transporters and over 200 MFS transporters have been revealed, with 100 MFS transporters more or less belonging to the multidrug resistance (MDR) proteins [16,52,53]. For example, 50, 44, and 54 genes were predicted to be ABC transporters in *Magnaporthe oryzae*, *Botrytis cinerea*, and *Fusarium* spp., respectively [26,52,54]. A total of 229 putative MFS transporter genes and 44 putative ABC transporter genes were identified from the *Zymoseptoria tritici* IPO323 genome database available at the JGI database (www.jgi.doe.gov) [55]. 

Some ABC transporters have been functionally characterized from plant pathogenic fungi (Table 1), yet the role of most remains unknown. Studies of ABC transporters in plant pathogens are often focused on their involvement in the development of multi-fungicide resistance, such as the BcATRB and the BcATRD in *B. cinerea* [39,56], and the PMR1 and PMR5 in *Penicillium digitatum* [17,57]. In addition, some ABC transporters have been associated with plant pathogenesis, presumably due to the export of plant defense compounds [58] (Table 1). With regard to MFS transporters, they are generally less understood compared with ABC transporters. The constitutive overexpression of MFS genes in several fungi have been shown to cause MDR phenotypes, including the MgMFS1 in *Z. tritici* [16], the PdMFS1 in *P. digitatum* [18], and the BcMFSM2 in *B. cinerea* [15]. The deficiency of some MFS transporters was found to be linked with increased sensitivity to a variety of chemicals, confirming their roles in MDR. Similarly, MFSs in plant pathogens can secrete host-specific toxins, affecting plant pathogenesis [52] (Table 1).

### 2.3. Efflux Transporter-Mediated Mechanisms of Fungicides Resistance

In phytopathogenic fungi, the first MDR phenotype was described in *P. digitatum* isolates from lemon fruit, which showed resistance to DMI fungicides including triflumizole, fenarimol, bitertanol, and pyrifenox [41]. Thereafter, field-relevant resistance to multiple chemical classes of fungicides were found in isolates of *Z. tritici* [16], *B. cinerea* [15], *Oculimacula yallundae* [59], *Sclerotinia homoeocarpa* [40], and *Penicillium expansum* [60]. Although exact mechanism(s) of MDR remained unknown in some pathogens (e.g., *O. yallundae*), it was typically caused by the overexpression of certain efflux transporters. 

#### 2.3.1. Expression of Efflux Transporters 

The majority of efflux transporters are silent or weakly expressed in the absence of toxic compounds, but their expressions can be rapidly induced by the addition of those drugs or phytoalexins [8]. Further, fungal sensitivity to antifungal agents could increase significantly without functional efflux transporters [61]. Although the expression of efflux transporters could be induced in as short as 10 to 15 minutes, this “lag period” seems to be sufficient for toxins to diffuse into fungal cells, leading to growth inhibition. However, if the transporters regulating fungal sensitivity are expressed constitutively, the drug uptake would be blocked and the resistance is thus conferred [20]. 

The constitutive overexpression of ABC transporter BcATRB and MFS transporter BcMFSM2 causing MDR phenotypes has been discovered in *B. cinerea*. Specifically, overexpression of BcATRB was found to confer resistance to fludioxonil, carbendazim, cyprodinil, and tolnaftate, and the associated phenotype was termed MDR1, whereas the phenotype displaying resistance to iprodione, boscalid, tolnaftate, cyprodinil, fludioxonil, fenhexamid, tebuconazole, bitertanol, and cycloheximide, caused by overexpression of BcMFSM2, was defined as MDR2 [15]. In addition, the natural hybridization of MDR1 and MDR2 was also revealed, designated as the MDR3 phenotype, which showed a combined resistance profile of MDR1 and MDR2 phenotypes [15]. Later on, a stronger MDR1 phenotype strain, MDR1h, with a higher expression level of the BcATRB was found to have more resistance to fludioxonil and cyprodinil than the MDR1 strains [62]. In *Z. tritici*, the causal agent of Septoria leaf blotch on wheat, overexpression of the major facilitator gene MgMFS1 was linked to strong resistance towards DMIs, and weak resistance towards QoIs and SDHIs [63,64]. Disruption of the *Mgmfs1* gene increased its sensitivity to several chemicals, including tolnaftate, epoxiconazole, boscalid [16], several QoIs, and an unrelated compound cercosporin [65]. In *P. digitatum*, overexpression of PMR1, PdMFS1, and PdMFS2 was correlated with complete or partial resistance to triflumizole [41], imazalil [66], and prochloraz [67]. In *S. homoeocarpa*, reduced propiconazole sensitivity was identified from five New England sites during a 2-year field efficacy study, with the EC_50_ values greater than 50-fold compared with those of the sensitive isolates. The overexpression of the PDR (pleiotropic drug resistance) transporter ShATRD was found to be strongly correlated with practical field resistance to propiconazole via transcriptomic and molecular analyses. Overexpression of the target gene of DMIs, ShCYP51b, only presents a minor factor affecting the sensitivity [40]. In addition to ShATRD, the overexpression of ShPDR1 is also correlated with practical field resistance to DMI fungicide propiconazole and reduced sensitivity to dicarboximide iprodione and SDHI fungicide boscalid [68]. 

#### 2.3.2. Modulator

Compounds able to modulate the activity of ABC or MFS transporters may reverse MDR, due to their inhibitory activity towards drug efflux from cells. Such compounds are usually called “modulators” or “inhibitors”. Among them, some modulators have been tested for their activity in plant pathogenic fungi. For *B. cinerea*, the phenothiazine chlorpromazine and the macrolide tacrolimus have modulator activity towards oxpoconazole [69]. In MDR2 and/or MDR3 isolates, synergism was found between modulator verapamil and tolnaftate, fenhexamid, fludioxonil, or pyrimethanil, suggesting that verapamil may inhibit the MFS transporter BcMFSM2 [70]. Similar inhibitory activity of verapamil, amitriptyline, and chlorpromazine toward the efflux was also observed in *Z. tritici* displaying MDR phenotype [16]. Whether modulators could be implemented for improving fungicide efficacy under field conditions needs to be further investigated.

### 2.4. Genetic Factors Underlying Overexpression of Efflux Pumps

#### 2.4.1. Transcription Factors

Two different mechanisms have been attributed to the overexpression of efflux pumps that are responsible for fungicide resistance. The most common one is amino acid changes in certain transcription factors that escalate expression levels of efflux pumps. As noted above, all *B. cinerea* isolates with MDR1 phenotype possessed point mutations in the MRR1, a transcriptional factor of BcATRB [15]. The mutations transform the transcription factors from a drug-inducible state to a permanently active state, resulting in the overexpression of an ABC transporter BcATRB. Transformation of the MDR1-type MRR1 confirmed that those point mutations are responsible for permanent activation of MRR1 and overexpression of BcATRB [15]. Furthermore, in MDR1h isolates, a deletion of 3-bp resulting in loss of amino acid (L^497^) in MRR1 caused overexpression of BcATRB than MDR1 isolates. In *S. homoeocarpa*, a gain-of-function mutation (M853T) in the activation domain of ShXDR1 renders constitutive overexpression of ABC transporters (ShPDR1 and ShATRD) and several CYP450 genes (CYP561, CYP65, CYP68), leading to the MDR phenotype [71]. However, expression of ATRB or other ABCs may not be exclusively regulated by a single transcription factor (e.g., MRR1) [72].

Interestingly, both MRR1 and ShXDR1 contain a Zn_2_Cys_6_ domain. Similarly, Wang et al. showed that MoIRR encoding a Zn_2_Cys_6_ transcription factor is associated with resistance in *M. oryzae* to isoprothiolane, a dithiolane fungicide used for rice blast control [73]. Mutations including R343W, R345C, and a 16-bp insertion in MoIRR were found in three lab mutants of *M. oryzae* with a moderate level of isoprothiolane resistance. In addition, cross-resistance with iprobenfos was observed, indicating that MoIRR may pay a significant role in resistance to choline biosynthesis inhibitors [73]. However, whether MoIRR regulates efflux pump(s) and whether those mutations may lead to differential expressions of other genes in the same mutants is largely unknown [74]. Transcription factors or activators containing fungal-specific Zn_2_-Cys_6_ DNA-binding domain were also found to contribute to DMI fungicide resistance in *Rhynchosporium commune* [75]. Apart from Zn_2_Cys_6_, leucine zipper transcription factors CaBEN1 and YAP 1, and zinc finger CRZ 1 have been involved in resistance or reduced sensitivity to benomyl in *Colletotrichum acutatum* [76], to several fungicide groups (i.e., clotrimazole, fludioxonil, vinclozolin, and iprodione) in *Alternaria alternata* [77], and to DMIs in *P. digitatum* [78], respectively. Collectively, these examples suggest that transcription factors may play a critical role in mediating fungicide resistance via regulating drug efflux pumps in most cases. 

#### 2.4.2. Promoter Rearrangement 

The insertion in the promoter region of an efflux transporter has also been reported to cause MDR. In *B. cinerea*, two types of rearrangements in the promoter region of BcMFSM2 were correlated with multidrug resistance. In the type A rearrangement, the BcMFSM2 promoter contained a 1326-bp insertion in conjunction with a 678-bp deletion. The type B rearrangement contained a 1011-bp insertion and a 76-bp deletion [79]. The MDR2 and MDR3 strains with the type A insertion occurred in the French (in Medoc, Champagne, Alsace regions), and German Wine Road regions, while the type B insertion was only found in the champagne region of France. MDR2 isolates harboring either the type A or type B rearrangement showed the same resistance phenotypes, with similar levels of BcMFSM2 overexpression [79]. Constitutive activation of the BcMFSM2 promoter by the rearrangement was confirmed by using reporter gene fusions [15]. Similarly, three different types of insertion (types I, II, or III, depending on the length of the insertion in the MFS1 promoter) have been reported to be present in the promoter region of the MgMFS1 promoter in *Z. tritici*. Through gene replacement, the insertions were verified to be responsible for MgMFS1 overexpression and the MDR phenotype [64]. The type I insertion was a long terminal repeat (LTR)-retrotransposon that could drive MgMFS1 expression by itself, as LTR elements typically contain cis-regulatory sequences. The type II insertion also possess similar upstream activation sequences (UASs), while the type III insert seemed devoid of regulatory elements. All those insertions resulted in the overexpression of MgMFS1, and thus cause MDR [64]. 

## 3. Standing Genetic Variation

Standing genetic variation typically refers to the presence of alternative forms of a gene (i.e., alleles) at a given locus in a population [80]. When environmental conditions change, the alternative allele may become beneficial. Thus, in addition to de novo mutations that subsequently sweep through the population, a fungal pathogen can possibly adapt to a new fungicide using alleles originating from the standing genetic variations [81]. In this review, heteroplasmy was also considered in the broad context of standing genetic variation. Up to date, resistance attributed to allele or gene variants is mostly documented for DMI fungicides, while the heteroplasmy phenomenon was found to be associated with resistance to QoI fungicides.

### 3.1. Paralogs of CYP51 and Its Mediated DMI Sensitivity 

CYP51, as the target of DMI fungicides, mediates a critical step of the synthesis of ergosterol, which is a fungal-specific sterol [82]. Multiple CYP51 paralogs have been found in ascomycete fungi. For example, two paralogs, CYP51A and CYP51B, were found in some *Aspergillus* species [83], *Colletotrichum* species [84], and *M. oryzae* [85], while *Fusarium* species possess three CYP51 paralogs [86]. Among CYP51s, CYP51B is considered the most conserved across ascomycetes, and CYP51A has been lost from multiple lineages [86,87]. It is noteworthy that a single paralog is typically not essential for fungal growth or infection when multiple paralogs exist, and only simultaneous inactivation of all paralogs is lethal [82,88]. 

Because protein structures are somewhat variable among CYP51s, the binding affinity of specific DMIs to the protein may therefore vary, which in term can result in selection for resistance in some populations with off-target CYP51 variants. Liu et al. showed that sensitivity to more DMI fungicides increased in CYP51A deletion mutants than CYP51C mutants, while no change in DMI sensitivity was observed for CYP51B deletion mutants [89]. Similarly, disruption of CYP51A in *A. fumigatus* [90] and *F. graminearum* [91] led to increased DMI sensitivity. Interestingly, Hawkins et al. showed that the re-emergence of CYP51A in the barley pathogen *R. commune* is responsible for resistance to various DMIs [87], and that the link between resistance and presence of CYP51A was observed in the global populations [92]. In some cases, CYP51 variants present in the same species could both mediate DMI sensitivity. Chen et al. reported that the disruption of CYP51A led to increased sensitivity to eight of nine DMIs tested in both *C. fioriniae* and *C. nymphaeae*, while disruption of CYP51B made mutants increasingly sensitive to five DMIs, suggesting species-specific, differential binding of DMI fungicides onto the two CYP51 enzymes. However, mutants expressing CYP51B largely had similar sensitivities to all fungicides tested regardless of species, suggesting a greater impact of CYP51A on DMI sensitivity due to its less conserved nature [84]. 

It was believed that CYP51A/B may have undergone a neofunctionalization process, where CYP51A gains novel functions through gene duplication and diversification while CYP51B retains the ancestral function [93]. As noted, CYP51 paralogs have been found in some fungal pathogens, for which use of DMIs may present a challenge. For instance, multiple *Colletotrichum* spp. are oftentimes involved in anthracnose disease in the same crop [2], where both paralogs especially CYP51A can be highly diverse, causing differential sensitivity among the species to a wide range of DMIs [94,95]. However, interestingly, mixing DMIs with different blinding affinities onto the two paralogs can result in synergistic effects [84], shedding light on the novel use of DMI fungicides.

### 3.2. QoI Resistance due to Mitochondrial Heteroplasmy 

Unlike nuclear genomes, multiple types of mitochondrial genomes can exist within a cell, termed mitochondrial heteroplasmy. This has been widely demonstrated in many eukaryotes that include fungi [96], and may confer a selective advantage under constantly changing environmental conditions [97]. QoI fungicides act to block the electron transfer in the respiration process, via binding the outer quinone oxidizing pocket of the cytochrome bc_1_ enzyme complex, which is encoded by the mitochondrial cytochrome b (CYTB) gene [98]. Zheng et al. showed the CYTB heteroplasmy in the apple scab pathogen *Venturia inaequalis*, where QoI-resistant mutants had both the wild-type genotype (G143) and resistant genotype (A143), and mitochondria-containing wild-type CYTB returned to high frequency during just two rounds of cultivations in the absence of kresoxim-methyl [99]. The heteroplasmic CYTB was also found in the field population of *V. inaequalis* and a high relative abundance of the A143 allele (>60%) was linked to the isolates highly resistant to trifloxystrobin [100]. Similar results were also observed in some powdery mildew fungi [101]. In some cases, the CYTB gene could remain heteroplasmic even after several years of cultivation without fungicide treatment [102]. But the frequency of the resistance-conferring A143 allele could also be dependent on the fungicide selection pressure, implying fitness costs in individual isolates [103]. 

Despite that QoI resistance due to mitochondrial heteroplasmy has been documented in many plant pathogens, the relative abundance of the A143 required to become a resistant phenotype seemed to vary greatly between species. Moderate resistant *V. inaequalis* isolates rarely contained A143 more than 8%, and only the G143 allele was detected by regular PCR and Sanger sequencing [100]. In *Blumeria graminis*, the lowest frequency of A143 detected in resistant isolates tested was 24% [103]. In contrast, *Podosphaera xanthii* isolates could harbor as much as 60% resistant allele, yet were sensitive to QoIs [104]. More studies aiming to understand the correlation between resistance level and frequency of A143 in relation to fitness costs are needed. 

## 4. Detoxification 

Pesticides can be detoxified by oxidation, reduction, or hydrolysis to give modified functional groups that are conjugated for excretion or deposition [105]. Some studies have suggested a three-phase system of xenobiotic-induced transcriptional regulation of the enzymes in mammals and arthropods, including phase I metabolizing enzymes such as cytochrome P450s, cytochrome P proteins, and monooxygenases, phase II conjugating enzymes such as sulfotransferase and glutathione S-transferase, and the phase III secretion system that consists of transmembrane transporters such as ABC transporters [71,106,107]. 

There have been some cases where detoxification or resistance development involved enzymes of these phases, such as overexpression of ABCs or MFSs described above. Additionally, in the *Mycobacterium* sp. strain SD-4, a hydrolase gene MHEL (phase I enzyme) was found to be capable of degrading the carbendazim (MBC) fungicide. The site-directed mutation experiment subsequently demonstrated that both Cys16 and Cys222 in MHEL were critical during the hydrolysis of MBC [108]. In *Botrytis pseudocinerea*, a cytochrome P450 monooxygenase CYP684 (phase I enzyme) was responsible for its natural resistance to the hydroxyanilide fungicide fenhexamid through oxidation. Interestingly, *B. cinerea* is highly sensitive to fenhexamid, although CYP684 is present in its genome that is closely related to *B. pseudocinerea*. Overexpression of CYP684 in *B. pseudocinerea* was revealed, presumably due to a 25-bp deletion in the promoter or a 10-bp insertion in the 3´ UTR of the gene [109]. In fact, P450-mediated resistance is considered a common type of metabolism-based resistance to insecticides [110], and has been associated with resistance in *Pyricularia oryzae* to phosphorothiolate fungicides [111]. For multi-site fungicides such as captan, reaction with non-essential thiol derivatives (phase II enzymes) has been suggested as a possible mechanism for detoxification [112]. More recently, Sang et al. reported that overexpression of phase I cytochrome P450s and phase III ABCs was regulated by a gain-of-function mutation of the transcription factor ShXDR1 in MDR isolates of *S. homoeocarpa* [71]. In this case, it could also be regarded as a drug efflux pump involved mechanism, although the extent to which the ABCs contributing to MDR phenotype was not clear. A better understanding of interaction between different detoxification phases in fungi will allow for an improved classification of resistance mechanisms. 

## 5. Regulation of Stress Response Pathways 

The members of group III hybrid histidine kinases (HHK) function as osmosensors in the high osmolarity glycerol (HOG) pathway that regulates response to environmental stress in fungi [113]. Because HHKs are not found in humans, they are considered an excellent molecular target for fungicides [114]. Phenylpyrroles, including fludioxonil and fenpiclonil, are derivates of the natural product pyrrolnitrin and have been widely used for disease control on many crops [115]. Phenylpyrroles have been found to interfere with the HOG pathway [116]. However, the target of phenylpyrroles is not very clear. Lawry et al. [117] demonstrated that fludioxonil failed to induce intact DRK 1 (a group III HHK from *Blastomyces dermatitidis*), to dephosphorylate its downstream target YPD1 in vitro, which would otherwise constitutively activate HOG signaling and cause cell death in vivo. This suggested that fludioxonil treatment may act on an upstream target that triggers HHK to become a phosphatase, which dephosphorylates YPD1 [117]. The same group later reported that fludioxonil interferes with triosephosphate isomerase (TPI), causing release of methylglyoxal (MG). The elevated MG likely in turn alters DRK1 activity via converting the kinase to a phosphatase that dephosphorylate Ypd1 to activate the HOG pathway and cell death [118]. Nevertheless, it has been found that mutations in several osmotic sensitivity loci such as OS1, OS2, and OS5 can cause resistance to fludioxonil and increased sensitivity to high osmolarity in different fungal pathogens [116,119,120,121], suggesting that certain fully functional loci in the HOG pathway may be essential for both the fungicidal effect and osmoregulation [116,120]. Bohnert et al. [122] identified and characterized a HOG1p-interacting phosphatase gene MoPTP2 in *M. oryzae* causing a rice blast, which can confer resistance to fludioxonil via overexpression. Similar to phenylpyrroles, dicarboxamide fungicides seem to also interfere with HOG pathway, and mutations in the same osmotic sensitivity loci oftentimes lead to dual resistance to both dicarboxamides and phenylpyrroles [116,119,120,123].

Fludioxonil is primarily used for *Botrytis* control in many small fruits and vegetables. Interestingly, field isolates showing resistance were mainly caused by mutations in MRR1 that regulates ATRB expression [62,72,124], despite that OS1 mutation(s) confer much higher level of resistance [119]. In fact, the os1 mutations were typically found responsible for fludioxonil resistance in laboratory mutants that had fitness costs compared to wildtype [119,125,126]. In contrast, the MDR phenotype of *Botrytis* isolates, conferred by overexpression of ATRB, dominated the population in a blackberry field over three years without fungicide selection pressure [127].

## 6. Other Alternative Resistance Mechanisms

Additional but less common non-target site mechanisms have also been reported, with most described for specific chemical groups or rather involving multiple genetic loci. For example, QoI resistance could also be associated with alternative oxidase (AOX). This is because the inhibition of the core pathway by QoIs may trigger the synthesis of alternative oxidase (AOX) to allow electrons from ubiquinol to bypass complex III, thus providing a QoI-insensitive pathway for oxidation of NADH [128]. Previous studies have suggested that AOX may contribute to QoI-resistance in several plant fungal pathogens [129,130,131,132,133,134]. A comprehensive review is also available regarding the role of AOX and its interaction with QoIs [128]. While alternative respiration can counteract the effect of QoIs, it would provide only 40% of the normal efficiency for ATP synthesis, due to the fact that complexes III and IV in the core pathway are bypassed and AOX lacks proton pump activity [135], which may be insufficient to support the ATP consumption during spore germination and host penetration. This implies that QoIs would be most effective against fungi during the pre-infection stage. Once infection has been established, the activation of the alternative pathway may significantly impact the efficacy of QoIs. Similar metabolic circumvention has also been associated with resistance in basidiomycete fungus *Ustilago maydis* and yeast to DMI fungicides, where altered sterol metabolism was observed to overcome the block of 14α-de-methylase and support fungal growth [136,137,138]. 

Unlike qualitative factors contributing to fungicide resistance where mutations are typically identified in single loci, multiple loci mutations have also been correlated with resistance. Examples include resistance to anilinopyrimidine (AP) fungicides in *Botrytis* isolates, in which nine individual genes involved in mitochondrial processes seemed to contribute to resistance, with BcMDL1 carrying the E407K mutation and BcPOS5 carrying the L412F mutation as the major factor [139]. Another example is DMI resistance in *Phaeosphaeria nodorum* isolates, where 34 candidate loci, including the target CYP51, were found in the genome, underlying quantitative variation in DMI sensitivity across populations [140]. Similar results were also found in the barley scald pathogen *R. commune*, which demonstrated that highly conserved genes *yvc1*, *ta*, and SDH made significant contributions to fungicide resistance in addition to CYP51A [75]. Furthermore, two loci that possibly contain a large number of transposon-related sequences were found to be associated with mefenoxam sensitivity in *Phytophthora infestans* [141]. Two non-target recessive genes in *Phytophthora capsici* were also believed to confer resistance to zoxamide [142]. 

**Figure 2:**
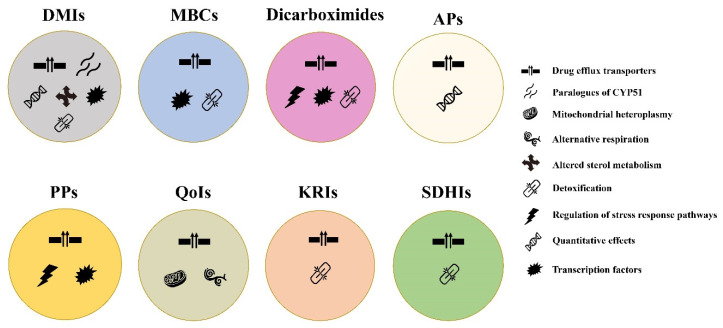
Non-target site mechanisms of resistance to major classes of fungicides used for crop disease management. Each circle with different colors represents a chemical class, whereas symbols within circles each represent a resistance mechanism as shown in the figure. DMIs: Demethylation inhibitors; MBCs: Methyl benzimidazole carbamates; APs: Anilino-pyrimidines; PPs: Phenylpyrroles; QoIs: Quinone outside inhibitors; KRIs: Ketoreductase inhibitors; SDHIs: Succinate dehydrogenase inhibitors.

The complexity of genomic pathways in diverse fungal populations has allowed them to respond and adapt to fungicides in multiple ways. Overall, to the best of our knowledge, there are at least nine non-target site mechanisms that can cause resistance to various chemical classes of fungicides (Figure 2). Remarkably, resistance caused by increased activity of drug efflux pumps has been characterized in a wide range of fungal pathogens for all major chemical classes, indicating its common role in resistance evolution. Other above-mentioned mechanisms such as detoxification, stress response pathway, and transcription factors have also been found for at least one chemical class. Evolution of DMI resistance seems to be the most diverse, presumably due to its long and frequent use at both agricultural fields and clinics [3]. 

## 7. Notable and Novel Mechanisms of Antifungal Resistance in Human Pathogens 

Use of fungicides in both clinics and agricultural fields has resulted in similar adaptations across phytopathogenic and clinical fungi over time, with the same or similar key resistance mechanisms such as target-site mutations, enhanced efflux pump activity, and activation of stress response pathways [3,143]. Given such similarity in evolution of fungicide resistance, our intent under this section was to highlight unique and novel molecular bases characterized in clinical fungi, to shed light on the exploration of unknown mechanisms of resistance in plant fungal pathogens. 

### 7.1. Acquisition of Resistance via Epigenetic Mechanisms 

Stability of fungicide resistance has been a key component in understanding fungicide resistance, especially when a new resistance is detected in a given fungus. While multiple mechanisms, as mentioned above, have been identified underlying stable resistance in most cases, mechanisms of transient or unstable resistance have yet to be described in phytopathogenic fungi despite such phenotypes or phenomena being commonly encountered under both lab and field conditions [144,145]. One possible mechanism of transient resistance is RNAi-based epimutation which was first discovered in the basal human fungal pathogen *Mucor circinelloides* with resistance to the antifungal agent FK506 [146]. The drug target peptidylprolyl isomerase FKBP12 was shown forming a complex to inhibit calcineurin. Intriguingly, RNAi was found to be spontaneously triggered to silence the FKBA gene encoding FKBP12, leading to the loss of the drug target, and thus the acquisition of resistance. These FK506-resistant epimutants were found to readily revert to a sensitive phenotype following several passages on FK506-free medium. Further, a reverted strain yet again was able to yield resistant epimutants at the same frequency [146]. Remarkably, a similar epigenetic mechanism was found to confer transient resistance to another chemical class of antifungal drug 5-fluoroorotic acid (5-FOA) by silencing the PYRF or PYRG encoding enzymes in the pathway of pyrimidine biosynthesis that converts 5-FOA into the active toxic form [147]. These novel findings indicate that RNAi-based epimutation may serve as an important mechanism for fungal pathogens to adapt to antifungal agents. A detailed review regarding epimutation-mediated drug resistance can be found in Chang et al. [10], which also covered chromatin-based forms of epigenetic resistance that described in *C. albicans* and other human pathogens. 

### 7.2. Mutator Genotype Accelerates Resistance Adaptation 

Genetic mutation plays a key role in the development of resistance to antifungal agents or antibiotics, and phenotypes exhibiting elevated mutation frequencies could therefore lead to higher rate of emergence of resistance [148]. However, because mutations can have lethal consequences, organisms have a range of repair systems to maintain their genome fidelity and stability. Thus far, two major DNA repair pathways in fungi including methyl-directed mismatch repair (MMR) and double-strand break repair (DSBR) have been associated with the mutator phenotype in which the rate of spontaneous mutation is greatly elevated [149]. Healey et al. [150] reported that a mutation or disruption of MSH2, an MMR gene, led to an increased rate of emergence of multiple antifungal resistance in *Candida glabrate*. It is noteworthy that these mutator phenotypes facilitated resistance development in an indirect way that led to increases in resistance-conferring mutations. For example, the MSH2Δ strain yielded a higher frequency of resistant offspring to echinocandin and all of those resistant MSH2Δ colonies had a mutation in either FKS1 or FKS2, which represents the primary mechanisms of resistance to this chemical class of antifungal drugs [150]. Similar mutator phenotype was also observed in *Cryptococcus neoformans*, in which the elevated rates of mutations were attributed to MMR genes MSH2, MSH5, and RAD5 [151].

Development of multifungicide resistance in fungi is believed to be via independent rounds of selection, which is supported by accumulation of mutations in the target genes causing resistance to respective fungicides [152,153,154]. Interestingly, there are solid field-evidences that showed a predisposition to selection for resistance in isolates that were already resistant to an unrelated fungicide [155,156]. This phenomenon implies that fungi might not only be selected for resistance, but also for an increased genetic plasticity that enables accelerated resistance development. Nevertheless, future studies aimed to understand correlations between mutator genotypes and multifungicide-resistant phenotypes are of great interest, likely revealing biological mechanism(s) underlying stepwise accumulation of resistance-conferring mutations in individual isolates. 

## 8. Conclusions

The structural change of fungicide targets caused by amino acid mutations typically confers much higher levels of resistance compared to other mechanisms, including those described above. However, there are many cases where fungicides fail to effectively manage crop diseases due to the emergence of resistance that is not linked to target sites, indicating the need for further understanding the diversity of resistance mechanisms. Thus, these non-target site mechanisms are not newly emerging threats but rather understudied. While a few mechanisms described above such as altered sterol metabolism have only been described and characterized in lab mutants, many others such as enhanced expression of drug efflux pumps and mutations in transcription factors have been widely reported in field resistant isolates. Nevertheless, future studies aiming to explore molecular bases of fungicide resistance, and assessing fitness costs and inheritance of non-target site-based resistance in a wide range of fungal pathogens may offer more insights into their impact on crop disease management.

## Figures and Tables

**Table 1:** ABC and MFS transporters identified in plant pathogenic fungi ^a^.

Species	Host	Protein	Group	Function	Fungicide Substrate	Reference
*Fusarium culmorum*	Cereals	FcABC1	ABC-G	Virulence, fungicide sensitivity	DMIs	[22,23]
*Fusarium graminearum*	Wheat	FgABCC9	ABC-C	Fungicide sensitivity; growth and pathogenicity	DMIs	[24]
*F. graminearum*	Wheat	FgARB1	ABC-F	Penetration; infection; growth	n.d.	[25]
*F. graminearum*	Wheat	FgABC1	ABC-C	Secretion of fungal secondary metabolites	n.d.	[26]
*F. graminearum*	Wheat	FgABC3	ABC-G	Virulence, fungicide sensitivity	DMIs	[26]
*F. graminearum*	Wheat	FgATM1	ABC-B	Iron homeostasis	n.d.	[27]
*F. graminearum*	Wheat	FgABC1 (FGSG_04580)	ABC-G	Virulence	n.d.	[28]
*Fusarium sambucinum*	Potato	GpABC1	ABC-G	Virulence	n.d.	[29]
*Nectria haematococca*	Pea	NhABC1	ABC-G	Virulence	n.d.	[30]
*Grosmannia clavigera*	Pine trees	GcABC1	ABC-G	Virulence	n.d.	[31]
*Magnaporthe oryzae*	Rice	MoABC1	ABC-G	Pathogenicity	n.d.	[32]
*M. oryzae*	Rice	MoABC2	ABC-G	Multidrug sensitivity	Blasticides, DMIs, antibiotics	[33]
*M. oryzae*	Rice	MoABC3	ABC-B	Infection structure formation	n.d.	[34]
*M. oryzae*	Rice	MoABC4	ABC-A	Pathogenicity	n.d.	[35]
*M. oryzae*	Rice	MoABC5	ABC-C	Pathogenicity	n.d.	[36]
*M. oryzae*	Rice	MoABC6	ABC-C	Hyphal growth	n.d.	[36]
*M. oryzae*	Rice	MoABC7	ABC-C	Conidiation	n.d.	[36]
*Botrytis cinerea*	Fruits, vegetables	BcATRA	ABC-G	Multidrug transporter	Cycloheximide, catechol	[37]
*B. cinerea*	Fruits, vegetables	BcATRB	ABC-G	Multidrug sensitivity	PPs, MBCs, APs, tolnaftate	[15]
*B. cinerea*	Fruits, vegetables	BcMFSM2	MFS	Multidrug sensitivity	Dicarboximides, SDHIs, tolnaftate, APs, PPs, KRIs, DMIs, cycloheximide	[15]
*B. cinerea*	Fruits, vegetables	BcMFS1	MFS	Multidrug sensitivity	DMIs	[38]
*B. cinerea*	Fruits, vegetables	BcATRD	ABC-G	Fungicides sensitivity	DMIs	[39]
*Sclerotinia homoeocarpa*	Turf grass	ShATRD	ABC-G	Fungicides sensitivity	DMIs	[40]
*Penicillium digitatum*	Citrus	PMR1	ABC-G	Fungicides sensitivity	Azole	[41]
*P. digitatum*	Citrus	PMR5	ABC-G	Multidrug sensitivity	MBCs, quinone, resveratrol, camptothecin	[17]
*P. digitatum*	Citrus	PdMFS1	MFS	Fungicides sensitivity; virulence	DMIs	[18]
*Zymoseptoria tritici*	Wheat	MgATR1	ABC-G	Transportation of various chemicals	DMIs	[42]
*Z. tritici*	Wheat	MgATR2	ABC-G	Transportation of various chemicals	DMIs	[42]
*Z. tritici*	Wheat	MgATR4	ABC-G	Virulence factor that affects colonization, fungicides sensitivity	DMIs	[43]
*Z. tritici*	Wheat	MgATR7	ABC-G	Iron homeostasis	n.d.	[44]
*Clonostachys rosea*	Soil-borne saprotroph	CrABCG29	ABC-G	H_2_O_2_ tolerance	n.d.	[45]
*Colletotrichum gloeosporioides*	Apple	CgABCF2	ABC-F	Asexual and sexual development; appressorial formation; plant infection	n.d.	[46]
*Colletotrichum higginsianum*	*Arabidopsis thaliana*	ChMFS1	MFS	Hyphal morphology; conidiation; pathogenicity	n.d.	[47]
*Colletotrichum acutatum*	Hot pepper	CaABC1	ABC-G	Conidiation; abiotic stress; multidrug sensitivity	Phosphorothiolates, QoIs, MBCs	[48]
*Gibberella pulicaris*	Potato	GpABC1	ABC-G	Virulence	n.d.	[29]
*Alternaria alternata*	Citrus	AaMFS19	MFS	Cellular resistance to oxidative stress and fungicides	Clotrimazole, PPs, inorganics	[49]
*Cercospora nicotianae*	Tobacco	CTB4	MFS	Virulence	n.d.	[50]
*Monilinia fructicola*	Peach	MfABC1	ABC-G	Fungicides sensitivity	DMIs	[51]

^a^ According to previous publications. DMIs: Demethylation inhibitors; MBCs: Methyl benzimidazole carbamates; APs: Anilino-pyrimidines; PPs: Phenylpyrroles; QoIs: Quinone outside inhibitors; KRIs: Ketoreductase inhibitors; SDHIs: Succinate dehydrogenase inhibitors; n.d.: Not determined.

## Data Availability

Not applicable.

## Abbreviations

Note: specific genes are not included.

**Abbreviation**	**Definition**
ABC	ATP-Binding Cassette
AP	Anilinopyrimidine
DMIs	Demethylation Inhibitors
DSBR	Double-Strand Break Repair
FOA	Fluoroorotic Acid
HHK	Histidine Kinases
HOG	High Osmolarity Glycerol
LTR	Long Terminal Repeat
MBC	Methyl Benzimidazole Carbamates
MDR	Multiple Drug Resistance
MFS	Major Facilitator Superfamily
MG	Methylglyoxal
MMR	Methyl-directed Mismatch Repair
NDAH	Nicotinamide Adenine Dinucleotide
NBD	Nucleotide-Binding Domains
PDR	Pleiotropic Drug Resistance
PPs	PhenylPyrroles
QoIs	Quinone outside Inhibitor
SDHIs	Succinate Dehydrogenase Inhibitor
SNP	Single Nucleotide Polymorphisms
TPI	Triosephosphate Isomerase
TMD	Transmembrane Domain
UAS	Upstream Activation Sequences
UTR	Untranslated Region
